# Benefits and Challenges of Scaling Up Expansion of Marine Protected Area Networks in the Verde Island Passage, Central Philippines

**DOI:** 10.1371/journal.pone.0135789

**Published:** 2015-08-19

**Authors:** Vera Horigue, Robert L. Pressey, Morena Mills, Jana Brotánková, Reniel Cabral, Serge Andréfouët

**Affiliations:** 1 Australian Research Council Centre of Excellence for Coral Reef Studies, James Cook University, Townsville, 4811, Queensland, Australia; 2 The Marine Science Institute, University of the Philippines, Diliman, Quezon City, 1110, Philippines; 3 School of Geography, Planning and Environmental Management, University of Queensland, Brisbane, Queensland, Australia; 4 Sustainable Fisheries Group, Bren School of Environmental Science and Management and Marine Science Institute, University of California Santa Barbara, Santa Barbara, CA, 93106, United States of America; 5 National Institute of Physics, University of the Philippines, Diliman, Quezon City, 1110, Philippines; 6 Institut de Recherche pour le Développement, UMR9220 ENTROPIE, Nouméa, New Caledonia; Leibniz Center for Tropical Marine Ecology, GERMANY

## Abstract

Locally-established marine protected areas (MPAs) have been proven to achieve local-scale fisheries and conservation objectives. However, since many of these MPAs were not designed to form ecologically-connected networks, their contributions to broader-scale goals such as complementarity and connectivity can be limited. In contrast, integrated networks of MPAs designed with systematic conservation planning are assumed to be more effective—ecologically, socially, and economically—than collections of locally-established MPAs. There is, however, little empirical evidence that clearly demonstrates the supposed advantages of systematic MPA networks. A key reason is the poor record of implementation of systematic plans attributable to lack of local buy-in. An intermediate scenario for the expansion of MPAs is scaling up of local decisions, whereby locally-driven MPA initiatives are coordinated through collaborative partnerships among local governments and their communities. Coordination has the potential to extend the benefits of individual MPAs and perhaps to approach the potential benefits offered by systematic MPA networks. We evaluated the benefits of scaling up local MPAs to form networks by simulating seven expansion scenarios for MPAs in the Verde Island Passage, central Philippines. The scenarios were: uncoordinated community-based establishment of MPAs; two scenarios reflecting different levels of coordinated MPA expansion through collaborative partnerships; and four scenarios guided by systematic conservation planning with different contexts for governance. For each scenario, we measured benefits through time in terms of achievement of objectives for representation of marine habitats. We found that: in any governance context, systematic networks were more efficient than non-systematic ones; systematic networks were more efficient in broader governance contexts; and, contrary to expectations but with caveats, the uncoordinated scenario was slightly more efficient than the coordinated scenarios. Overall, however, coordinated MPA networks have the potential to be more efficient than the uncoordinated ones, especially when coordinated planning uses systematic methods.

## Introduction

International conservation policies have encouraged the formation of a global network of marine protected areas (MPAs) as one approach to mitigating the continuing decline of fisheries and marine ecosystems [1]. Ideally, systematic conservation planning (hereafter “conservation planning”) should be employed to create regional networks of MPAs to achieve ecological objectives [2]. Conservation planning is a spatially-explicit framework for designing and locating actions that promote biodiversity conservation and sustainable use of natural resources to help preserve ecosystem function and support human activities [3, 4]. This approach is promoted for selecting MPAs because, at least in principle, it is an efficient means to attain conservation objectives, incorporates diverse types of data, and can promote the participation of stakeholders [5, 6]. However, conservation planning has a poor track record of translating into local actions [7]. Initially, conservation planning was based on purely biophysical information [8], but efforts to incorporate socioeconomic considerations are increasing [9–11]. Beyond the considerations that have been made operational in conservation plans, successful implementation of protected areas still depends on numerous social, economic, and political factors, including institutional capacity and priorities, financial constraints, and tenure [12–14]. Moreover, management and governance of extensive marine systems is very complex and requires innovative approaches to link institutions across multiple scales [15].

The Coral Triangle region, formed by Indonesia, Malaysia, Papua New Guinea, Philippines, Solomon Islands, and Timor Leste, has complex marine governance, and very extensive and diverse marine ecosystems that are severely threatened by human activities [16–18]. Many of the MPAs in the region are small, locally-established, and locally-managed due to decentralization of government or customary marine tenure [16, 17, 19–24]. Some of the locally-established MPAs in the region have been effective at achieving local-scale fisheries and conservation objectives [17, 21, 25]. Examples of local-scale objectives are to maintain and/or increase the abundance and biomass of economically-important fish species and to maintain or improve habitat condition [26, 27]. These local MPAs can be relatively easy to implement because of the direct and tangible benefits to local communities, thereby increasing support from the people affected by the constraints on resource use [28, 29]. Community members are directly involved in the decision-making processes and management, which enables them to perceive the benefits from their initiatives [30–32].

Despite these successes, locally-established MPAs in the Coral Triangle are typically small (usually <1 km^2^) and were not intended to form ecological networks [21, 33]. These small MPAs might not contribute substantially to broader objectives such as maintaining larval connectivity to allow for population replenishment, and connectivity between habitats to support species that require different habitats at various ontogenetic stages [1, 31, 32]. Hence, small MPAs are assumed to be insufficient to contribute to the national, regional, and global networks of MPAs that are mandated by international policies [33]. There is a growing understanding of the need to match ecological scales with governance scales in the Coral Triangle [16]. For this to happen, conservation planning offers a framework to create regional designs that can be “scaled down” or used to guide local actions [34]. Alternatively, local actions can be “scaled up” or coordinated to address broader-scale conservation objectives [17].

Scaling up is one approach to bridge the gap between regional goals and local actions. It involves the expansion of local actions using coordinated and integrated approaches with a regional perspective. It entails widening the context for local decisions from smaller areas to larger areas (e.g. bays to seascapes) to address both local and broader-scale objectives, which requires involvement of more people and institutions [35–37]. In the context of forming MPA networks, coordinated expansion is defined here as establishing additional MPAs based on collaborative planning to address objectives across multiple governance units.

The Philippines offers good examples of scaling up MPAs. Scaling up locally-managed MPAs to form networks is done through collaborative initiatives such as the inter-local-government alliances (hereafter “alliances”). These alliances are formed amongst neighbouring local governments within a bay or fishing ground and are usually facilitated and supported by bridging organizations, including academe and non-government organizations. The formation of alliances is catalysed by the urgency and understanding of local governments’ needs to share responsibilities to address mutual problems, such as overfishing and pollution, that they cannot solve on their own. Experience in the Philippines is that the purposes of alliances evolve through time. Initially, the purpose of the alliances is to share experiences and activities for management of coastal resources (e.g. enforcement, awareness campaigns) and create mutual funds systems. As the alliances gain more experience, they are able to coordinate establishment of additional MPAs to form MPA networks. These MPAs can be larger than those established by single local governments, sometimes straddling municipal boundaries and addressing broader-scale objectives such as improved habitat representation and connectivity [36, 37].

While scaling up shows much promise in addressing regional objectives, evidence that demonstrates the benefits of coordinated establishment of MPAs is still lacking. Hence, in this paper, we used the Philippines as a case study to demonstrate and describe the benefits of coordinated establishment. We did this by building on the work of Mills, Adams [38], which compared the efficiency of the ad hoc, uncoordinated approach to MPA expansion with conservation planning for attaining specific objectives for habitat representation. They found that the uncoordinated approach was less efficient than conservation planning, achieving only half of the objectives for habitat representation over 10 years with the same rate of MPA expansion. Here, we add an intermediate dimension to the work of Mills, Adams [38], by considering the coordinated establishment of MPAs in the Philippines, and describing its benefits relative to both conservation planning and uncoordinated establishment of MPAs.

In this study, we aim to present how much benefit can be gained from coordinating MPA initiatives as compared to uncoordinated community-based establishment in terms of achieving objectives for habitat representation. Our specific goals are to:

- Simulate the expansion of MPA networks using seven different scenarios reflecting different levels of coordination, spatial contexts for governance, and approaches to planning;
- Determine the differences between scenarios in achieving objectives for habitat representation; and,
- Assess the potential advantages of coordinating initiatives as compared to both uncoordinated community-based efforts and conservation planning.

This study addresses a key knowledge gap in policy and practice for coastal and marine resource management in countries with small, disparate governance units, and complex social, economic, and political contexts for marine conservation.

## Methods

### Ethics statement

The first author had been given ethics approval (H3995) by Human Research Ethics Committee of James Cook University to conduct key informant interviews for this research project. Consent forms were signed by the informants who participated in the interviews. Some of the data used in this research project were copyright materials from Conservation International–Philippines (hereafter “CI-Philippines”). The first author had a licence agreement, which details the terms and conditions of the use of the copyright materials, with CI-Philippines. The licence agreement was signed by the previous director Mr. Romy Trono in August 2011, and will expire after publication of the research project.

### Policy context and study region

In 2006, the Philippines National Policy on Biological Diversity (Executive Order 578) identified the Sulu-Sulawesi Marine Ecoregion and the Verde Island Passage as national priorities for marine conservation. The Verde Island Passage is deemed to be at the “centre of the centre” of marine shore-fish diversity in the Indo-Malay-Philippines archipelago [39]. It is home to many marine species, including marine mammals, turtles, economically important species of pelagic and reef fish, corals, and other invertebrates. The Verde Island Passage is also subject to numerous threats such as overfishing, shipping, and pollution from upland agriculture and shoreline development and industries. The call to protect the Verde Island Passage has made it the model for building MPA networks in the Philippines [40]. For all these reasons, we used the Verde Island Passage as the planning region for this study.

**Fig 1:**
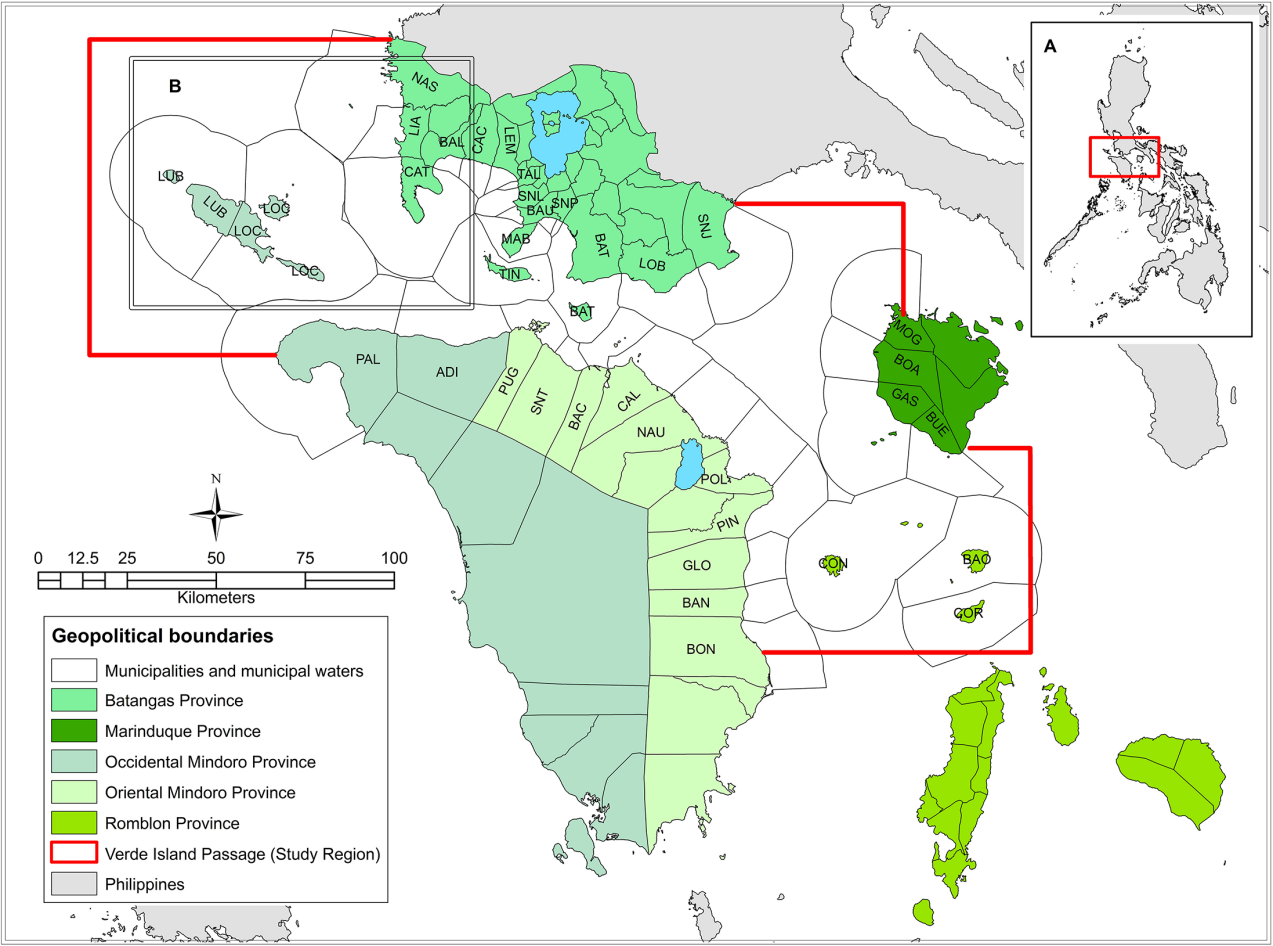
Geopolitical scales within the Verde Island Passage. The five provinces surrounding the study region are indicated by colours. Inset A. Location of the Verde Island Passage within the Philippines. Inset B. Area shown in detail in Fig 2 to illustrate the habitat mapping that covers the entire study region. The 36 coastal municipalities surrounding the Verde Island Passage are listed here by province. Batangas Province: NAS–Nasugbu, LIA–Lian, CAT–Calatagan, BAL–Balayan, CAC–Calaca, LEM–Lemery, TAL–Taal, SNL–San Luis, BAU–Bauan, MAB–Mabini, TIN–Tingloy, SNP–San Pascual, BAT—Batangas City, LOB–Lobo, SNJ–San Juan. Marinduque Province: MOG–Mogpog, BOA–Boac, GAS–Gasan, BUE–Buenavista. Occidental Mindoro Province: LUB–Lubang, LOC–Looc, PAL–Paluan, ADI–Abra de Ilog. Oriental Mindoro Province: PUG–Puerto Galera, SNT–San Teodoro, BAC–Baco, CAL–Calapan City, NAU—Naujan, POL–Pola, PIN–Pinamalayan, GLO–Gloria, BAN–Bansud, BON–Bongabong. Romblon Province–CON–Concepcion, BAO–Banton, COR–Corcuera.

**Fig 2:**
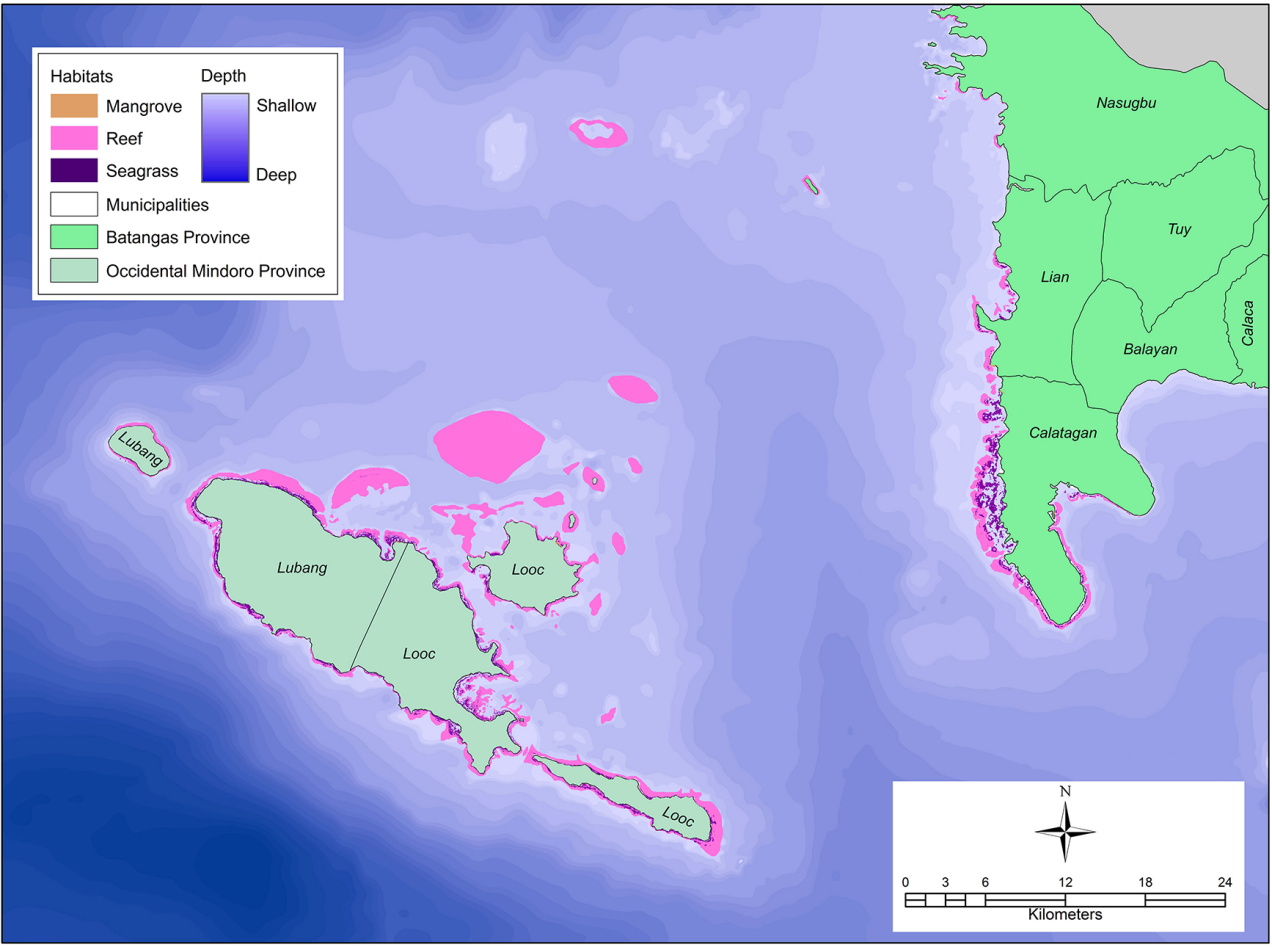
Habitat classification used in the scenarios. The Verde Island Passage typically has narrow fringing shallow-water formations with steep descents into deep water. The most extensive shallow portions of the region are shown in this figure, including the largest areas of coral reefs, seagrass, and mangrove habitats in the study region. These areas are surrounded by the municipalities of Nasugbu, Lian, and Calatagan in Batangas Province and the municipalities of Lubang and Looc in Occidental Mindoro Province.

Efforts to establish the Verde Island Passage MPA network have been made by CI-Philippines, together with the local governments responsible for the region’s coastal ecosystems. The region is surrounded by five provinces, namely: Batangas, Marinduque, Occidental Mindoro, Oriental Mindoro, and Romblon (Fig 1). It is characterized by generally narrow and fringing marine habitats, with very steep drop-offs in the centre of the Passage, such as the narrowest portion where Isla Verde is located, around the islands of Romblon and Marinduque, and in some coastal areas of Occidental Mindoro. The shallowest portions of the Passage are located in Batangas, and some portions of Lubang Island in Occidental Mindoro (Fig 2) [40].

The Verde Island Passage MPA network is currently administered by three clusters of collaborating local governments. The two provincial networks are the Batangas Province MPA and enforcement network and the Oriental Mindoro Province MPA and enforcement network. In these networks, enforcement teams assist MPA managers by coordinating patrolling activities and sharing information on illegal fishers in MPAs and commercial fishers encroaching on municipal waters. The enforcement teams are comprised of fisher volunteers, police, and coast guards. The third administrative cluster consists of the municipalities of Lubang and Looc in Occidental Mindoro, which have initiated collaborations to form their own MPA network within the Verde Island Passage.

Prior to the establishment of the Verde Island Passage network in 2008, around 30 uncoordinated locally-managed MPAs were established with a total area of 8.74 km^2^. Some of these MPAs were established with support from various bridging organisations, but did not involve consultation with neighbouring local governments. However, with assistance from CI-Philippines, alliances of local governments coordinated protection of around 1.5% (~170 km^2^) of the Verde Island Passage’s marine extent from 2008 to 2011. This total area consists of 69 established MPAs as part of the existing MPA network, and zoned as permanent no-take areas or permanent marine reserves where fishing is restricted only to hook and line. Most of these MPAs protect coral-reef habitats. Efforts to increase the number and extent of MPAs in the Passage, and to protect non-coral habitats, are underway to fulfil the targets for habitat representation urged by various international policies, such as the Convention on Biological Diversity (CBD) and Coral Triangle Initiative (CTI).

### Study design

**Fig 3:**
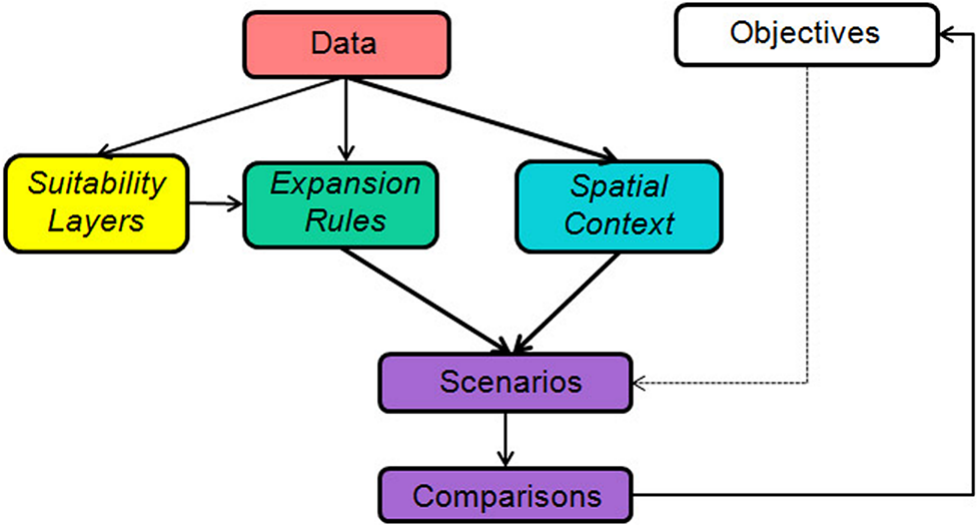
Study design. Scenarios were defined by combinations of spatial contexts, suitability layers, and expansion rules, and compared in terms of achieving objectives for representation of each mapped marine habitat.

We simulated seven spatially-explicit expansion scenarios, representing systematic and non-systematic planning approaches, of the MPA network in the Verde Island Passage (Table 1). These expansion scenarios were developed to demonstrate and compare their benefits in terms of achievement of objectives for habitat representation. Each expansion scenario was characterized by a combination of a suitability layer, expansion rules, and spatial context (Fig 3). Once all the simulations were run, we compared their achievement of conservation objectives for the study region at year 2020. We describe in the succeeding sub-sections how each of the expansion scenarios was developed.

**Table 1:** Detailed description of each MPA expansion scenario simulated in this study. Please refer to Tables (S1 and S2 Tables) and Figures (S1 and S2 Figs) for detailed information on the suitability layers and decision trees for the uncoordinated and coordinated scenarios.

Scenario	Description	Spatial context	Suitability layer	Expansion Rules	Conservation objectives
1. ***Non-systematic, uncoordinated*** MPA establishment undertaken by ***local governments individually***	MPAs were established either by communities and/or local governments independently, without guidance or with only minimal guidance from bridging organizations and without consideration of ecological processes across areas larger than municipalities. This depicted the situation prior to efforts to establish the Verde Island Passage MPA network. This situation could recur if efforts to sustain collaborative partnerships diminish.	***Municipal waters (individual municipalities)***: Territorial waters of local governments (within 15 km from the shore of each municipality) based on the Local Government Code.	***Uncoordinated***: Suitability for MPAs was based on the characteristics of the MPAs established before coordination began in 2008, and from key informant interviews. Factors used to determine suitability of planning units for MPA establishment include habitat types, accessibility, and distance to another MPA.	***Uncoordinated***: The decision tree used the suitability layer and spatial context to determine the location of potential MPAs. The MPA sizes from the database (prior to 2008) were used to inform the simulation for assigning sizes of MPAs.	Test if 20% of each habitat was protected in each municipality.
2. ***Non-systematic, partially coordinated*** MPA establishment undertaken by ***local government alliances***	Efforts to coordinate MPA planning and management were undertaken by alliances of local governments, each with one to five municipalities in a shared bay, gulf, or coastal stretch. Within alliances, local governments were collaborating to establish MPAs. Support was provided by bridging organizations to identify potential MPAs using ecological information about the region.	***Shared municipal waters across alliances***: Local governments in an alliance might have an agreement to jointly manage their municipal waters as recommended by the Fisheries Code.	***Coordinated***: Suitability for MPAs was based on the characteristics of areas where MPAs were established when coordination was initiated and facilitated by CI-Philippines from 2008 onwards. Factors used to determine suitability of planning units for MPA establishment included habitat types, fisheries importance, and land-based and coastal threats.	***Coordinated***: The decision tree used the suitability layer and spatial context to determine the location of potential MPAs. The MPA sizes from the database (from 2008–2010) were used to inform the simulation for assigning sizes of MPAs.	Test if 20% of each habitat was protected in shared municipal waters within each alliance.
3. ***Non-systematic, fully coordinated*** MPA establishment undertaken by ***local governments and their corresponding provincial governments***	Efforts to coordinate MPA planning and management were in place at the provincial level. Each provincial government was working with its respective local governments to schedule MPA establishment with support from bridging organizations using ecological information across the province.	***Shared municipal waters across provinces***: Local governments across provinces might have an agreement to jointly manage their municipal waters as recommended by the Fisheries Code.	***Coordinated*** as described above	***Coordinated*** as described above	Test if 20% of each habitat was protected in shared municipal waters within each province.
4. ***Systematic*** approach to MPA establishment in ***individual municipal waters***	This scenario involved establishment of MPAs by individual local governments with guidance from conservation planning software and ecological information about the Verde Island Passage. There was no coordination between municipalities.	***Municipal waters (individual municipalities)*** as described above for scenario 1	***Inverse of the uncoordinated suitability layer***: The inverse of the uncoordinated suitability layer was used as the cost layer to encourage protection of more suitable areas. Using the uncoordinated suitability layer allowed for comparison of areas protected with the uncoordinated community-based scenario (1).	***Marxan***: Maximise the achievement objectives while constraining establishment of MPAs based on the annual average rate of establishment during coordination.	Protection of 20% of each habitat in each municipality.
5. ***Systematic*** approach to MPA establishment facilitated by local government ***alliances***	This scenario involved establishment of MPAs by local government alliances with guidance from conservation planning software and ecological information about the Verde Island Passage.	***Shared municipal waters (alliances)*** as described above for scenario 2	***Inverse of the coordinated suitability layer*** to encourage protection of more suitable areas, and to allow comparison with the corresponding non-systematic scenario (2)	***Marxan*** as described above	Protection of 20% of each habitat in shared municipal waters within each alliance.
6. ***Systematic*** approach to MPA establishment facilitated by ***provincial networks***	This scenario depicted MPA establishment by provincial governments together with their local governments with guidance from conservation planning software and ecological information about the Verde Island Passage.	***Shared municipal waters (provinces)*** as described above for scenario 3	***Inverse of the coordinated suitability layer*** to encourage protection of more suitable areas, and to allow comparison with the corresponding non-systematic scenario (3)	***Marxan*** as described above	Protection of 20% of each habitat in shared municipal waters within each province.
7. ***Systematic*** approach applied to a ***regional MPA network***	This scenario depicted MPA network formation using conservation planning software, whereby the spatial boundaries of governance units within the Verde Island Passage region were not considered.	***Shared municipal waters (region)*** Boundaries of governance units were not considered in the VIP region.	***Inverse of the coordinated suitability layer*** as described above	***Marxan*** as described above	Protection of 20% of each habitat across the Verde Island Passage.

### Data, sources of information, and conservation objectives

We obtained spatial data for existing MPAs, resource uses, threats, habitats, and fisheries from CI–Philippines. These datasets were used to develop the suitability layers for additional MPAs in the expansion scenarios. We supplemented habitat maps from CI–Philippines with new maps of coral reefs from the Millennium Coral Reef Mapping Project [41]. The habitats considered in the analyses were coral reefs, mangrove forests, seagrass beds, and “other benthic substrata”, including rocky and soft-sediment seabed. For other benthic substrata, we used five depth classes from bathymetric maps (0–10 m, 10–20 m, 20–30 m, 30–40 m and >40 m) to reflect expected changes in species composition with depth [42–44]. We based our conservation objectives on the CTI—National Plan of Action [45] which recommended that 20% of the extent of each major coastal and marine habitat should be set aside in permanent no-take zones.

### MPA expansion scenarios

#### Governance context and areas

The Philippine national government does not have specific mandate describing the explicit distribution of MPAs to address objectives for biodiversity conservation. Although the national government has powers to establish MPAs, much of the responsibility for planning and management of natural resources has been devolved to local governments. The mandates of the Local Government Code (Republic Act 7160) and the Fisheries Code give local governments the task of establishing MPAs within their municipal waters. However, local governments can also share responsibilities and combine their efforts to co-manage resources in contiguous waters (e.g. shared bays, gulfs). We used this information to identify four governance contexts for expansion of MPA networks: 1) individual municipal waters (36 governance areas); 2) contiguous shared municipal waters in bays and coasts within provinces (10 areas); 3) contiguous shared municipal waters for all local governments within each province (5 areas); and, 4) the entire Verde Island Passage.

#### Defining scenarios

Each of the seven scenarios simulated the expansion of MPAs in the Verde Island Passage (Table 1). The first three scenarios described below represent non-systematic approaches to expansion; the remaining four scenarios represent systematic expansion. The uncoordinated scenario (1) depicts establishment of MPAs by communities or local governments in the waters of 36 individual municipalities. The partially coordinated scenario (2) involves coordination between two or more local governments within alliances (10 alliances). The fully coordinated scenario (3) involves coordination of all the local governments in each province with their corresponding provincial governments (5 provinces). The latter two scenarios reflect how collaborative partnerships are initiated and scaled up to higher governance levels in the Philippines [37].

The uncoordinated scenario was based on the MPA efforts initiated by individual municipalities and their corresponding communities prior to coordination (before 2008). The partially and fully coordinated scenarios were based on the existing institutional arrangements by the provincial and local-government alliances in the Verde Island Passage (2008 onwards). These scenarios were programmed using MatLab (R2011b) software (see text on expansion rules, below).

The four systematic scenarios were designed to reflect MPA establishment guided by explicit objectives in conservation planning software, but varied in the spatial contexts considered for objectives and selection of areas (Table 1). The spatial contexts for three systematic scenarios (4–6) matched those of the non-systematic scenarios (1–3) to allow direct comparisons, with the final scenario applying conservation planning across the whole Verde Island Passage.

The varying levels of coordination in scenarios 1–3 reflect different constraints on and opportunities for local governments to coordinate their planning and management of MPAs. Coordination can be constrained by logistical concerns, spatial extent, and availability of funds. Local governments closer together, such as those surrounding a single bay, will collaborate more readily because it is easier for them to communicate and share meetings, and some local governments work well together because of similar interests and goals [37, 46]. Coordinating efforts across larger spatial extents, regardless of the number municipalities, presents challenges. Although, there have been technological advances in communications, not all municipalities in the Philippines have been able to avail of these technologies to help them coordinate with neighbouring municipalities and other government agencies. Moreover, in-person meetings are still preferred and found more valuable by local-government officials and employees, compared to online or telephone communication. Hence, scaling up begins in alliances of two or more local governments. Coordination also depends heavily on funding. Lack of finances or lack of willingness to contribute to a mutual funds system strain relationships and lead to partial coordination [37]. We defined partial coordination as subject to logistical and spatial constraints. Hence, for our partial coordination scenario, alliances of two to five neighbouring local governments are formed, since they are easier to organize and mobilize due to their proximity. We defined full coordination as collaboration between all coastal local governments, across whole provinces, with support from provincial governments.

#### Planning units

For all scenarios, we subdivided the planning region into grids of 1 km^2^ planning units (n = 15,121), cut around existing MPAs, coastlines, and governance boundaries (municipalities, alliances, provinces). With this method, existing MPAs were single planning units. Depending on the scenario, we assigned each planning unit in the region to different governance areas, but retained the same number of units for all scenarios. Planning units contained one or more habitat types.

For the systematic scenarios, selected whole planning units were allocated to protection. In contrast, and to reflect the observed establishment of different sizes of MPAs, the decision trees for the non-systematic scenarios, below, selected parts of planning units. To match the reality of recent decision-making, varying proportions of selected planning units, always less than the full size of 1.0 km^2^, were protected in the uncoordinated scenario (1). For the coordinated scenarios (2 and 3), the varying proportions of planning units protected extended to 1.0 km^2^, reflecting the larger MPAs established through coordination. Both size distributions were implemented in the expansion rules, described below.

#### Suitability layers for the MPA expansion scenarios

For all scenarios, we modelled the suitability of planning units outside existing MPAs for potential establishment of new MPAs. Each planning unit had one value representing its suitability for MPA establishment.

The uncoordinated and coordinated suitability layers were shaped by different factors that determined the likelihood of planning units being selected as potential MPAs. The suitability of planning units for each scenario was based on spatial predictors derived from characteristics of existing MPAs and interviews with key informants (Table 2). Key informants were selected based on their understanding and experience working on MPAs in the region and in the Philippines. The informants included managers of community-based local MPAs, local-government officials, and MPA experts in universities and bridging organizations. Informants were asked to identify the criteria used for MPA establishment.

**Table 2:** Factors, decision rules, and spatial predictors used to inform the suitability layers for the uncoordinated scenario (US, scenario 1) and coordinated scenarios (CS, scenarios 2 and 3). √ indicates that the spatial predictor was used to create the suitability layer for the scenario(s).

Factors considered for the location & size of MPAs	Spatial predictors	Rationale explained by key informant interviews and scientific literature	US	CS
1. Establishment of MPAs by adjacent *barangays* or local government units	Distance from another MPA	MPAs tend to clump together in one area, since local governments interested in implementing MPAs tend to establish more than one MPA in their municipalities. Some municipalities in the country have one MPA in each village within their waters, provided that fishing communities were interested as well.	√	
2. Accessibility, visibility from barangay, and ability to enforce and monitor resource regulations	Distance from the shoreline	Even though municipal waters extend 15 km from the shoreline, most MPAs were established within 5 km of the shore for ease of enforcement. This enabled MPA guards to easily see violators and apprehend them, since most of the guards have only non-motorized boats. MPA guardhouses were set close to roads and near villages to allow ease of access and cheaper maintenance.	√	√*
	Distance from roads			
3. Habitat health, productivity and type; perceived benefit of implementing MPAs for tourism purposes apart from achieving fisheries objectives	Habitat type	Productive and healthy habitats were protected mostly to sustain biodiversity, abundance and biomass of flora and fauna, and reduce impacts of threats apart from fisheries and other human activities. However, habitats that were degraded were also protected to allow them to recover (e.g. mangrove rehabilitation). Data on habitat health were available only for the existing MPAs; hence habitat type was used as a surrogate. Coral reefs were protected mostly due to the potential added benefits of allowing access to certain zones of the MPAs for tourism purposes. Communities then have an added or alternative source of income by introducing user fees, serving as tour guides, and involvement in other tourism-related activities. Mangrove MPAs were also initiated, since they are potential areas for establishing boardwalks and paddle-boat tours whereby tourists can observe associated fauna (e.g. birds, reptiles, fireflies). Increasing representation will aid in maintaining connectivity within patches of the same habitat types (e.g. coral reefs to coral reefs; seagrass bed to seagrass bed) and between habitat types (e.g. mangrove to seagrass; coral reef to seagrass).	√	√
4. Shoreline development	Distance from developed areas and other threats	MPAs were not established in areas (e.g. ports and factories) most likely to be affected by human impacts. This was to avoid disturbance and allow recovery.		√
5. Marine threats	Presence of marine threats (e.g. illegal fishing)	Areas that are heavily fished are also protected since they are assumed to be important habitats or highly productive areas (e.g. coral reefs, upwelling areas for pelagic species).		√
6. Temperature refugia and larval entrainment potential	Temperature refugia (data not available)	Areas identified as temperature refugia should be protected to reduce threats that may affect them since they can provide propagules after reefs elsewhere have been bleached. Larval source and sink areas should be protected to maintain connections.		√
	Larval entrainment potential	Areas deemed to have high larval entrainment potential (based on icthyoplankton distribution, chlorophyll concentrations & larval dispersal modelling) should be protected since they can serve as good sources and sinks of larvae.		
7. Presence of threatened species and marine megafauna	Presence of threatened species and marine megafauna	Communities are now protecting turtle nesting sites and areas where dolphins, whales and whale sharks are sighted since they are seen as potential ecotourism sites, following the success of various whale shark interactions and whale watching tours.		√

*For the coordinated scenarios, we excluded distance from roads because patrolling was no longer limited in terms of access.

Motorized boats provided by Conservation International–Philippines, who supported and facilitated coordination of the local governments, improved patrolling and reduced road travel of MPA patroller.

We used Maxent to model the two suitability layers based on the spatial predictors (Table 2) identified by informants. Maxent was developed to predict the suitability of areas for species [47], but has characteristics that make it appropriate for modelling suitability for establishment of different kinds of MPAs [38]. Maxent uses presence-only data to predict areas of interest based on observed characteristics, and the input data on existing MPAs were presence-only. The modelled suitability layers were then incorporated into the expansion rules (discussed below) for selection of new MPAs.

The suitability layer for the uncoordinated scenario used spatial predictors derived from MPAs established by communities and single local-government authorities prior to coordination (before 2008). The four predictors were: a) distance from another MPA; b) distance from shoreline; c) distance from roads; and, d) habitat type [48–51] (Table 2). For the coordinated scenarios, we used the same spatial predictors that CI-Philippines used to select MPAs, including: a) distance from shoreline; b) habitat type; c) distance from shoreline development [52]; d) presence of marine threats; e) potential for larval retention [51, 53, 54]; and, f) presence of threatened species and marine megafauna (Table 2). We used Maxent to replace the manual application of individual criteria by CI-Philippines to a consistent, integrated, and quantitative suitability layer.

For the systematic scenarios, we developed cost layers for Marxan (Table 1), depending on the spatial context for selections of additional MPAs, as the inverse of the uncoordinated (scenario 4) and coordinated suitability layers (scenarios 5–7), thereby allocating lower costs and higher likelihood of selection to more suitable planning units. This allocation of cost layers also facilitated direct comparisons of systematic and non-systematic scenarios in three governance contexts (Table 1).

#### Expansion rules

We included all the existing MPAs as starting points for all the scenarios. The simulations required an annual rate of expansion of MPA networks, so we calculated the average annual area protected in the region. From 1991 to 2007, establishment of MPAs was opportunistic and done by individual local governments. MPAs during that time were very small with an annual average rate of expansion of 0.22 km^2^ for all MPA types. Additional, larger MPAs were established from 2008 onwards, with an annual area protected of approximately 82.8 km^2^ for all types of MPAs. This later rate of establishment reflects more recent efforts to coordinate MPA establishment and is the rate used for all seven scenarios.

All scenarios involved simulation of the expansion of MPA networks by establishing MPAs beginning in 2012 and ending until 2020. There are different types of MPAs established in the Philippines, based on the level of government involved (e.g. national, local), supporting legislation, and consultation with different stakeholder groups, particularly fishers. Locally-established MPAs usually have different zones such as fish sanctuaries (strictly no-take zones), marine reserves (regulated fishing zones), and fisheries management areas (temporal closures) [17]. Because we did not have data on the relative effectiveness of different MPA zones for protecting different species and habitat types, we assumed that all the MPA zones made equal contributions to conservation objectives. This provides an optimistic picture of achievement of conservation objectives during the simulations.

There were two decision trees for the three non-systematic scenarios (Table 1): one for uncoordinated decisions (scenario 1; S1 Fig, S1 Table) and one for coordinated decisions (scenarios 2 and 3, applied within different governance contexts; S2 Fig, S2 Table). The decision trees reflected approaches to establishing MPAs prior to (scenario 1) and during (scenarios 2 and 3) coordinated MPA expansion efforts. They were intended to reflect how local governments, MPA managers, and their corresponding communities decide on the locations and sizes of MPAs within each spatial context. The decision trees used information on existing MPAs, policy information, and interviews with key informants. Information on existing MPAs guided the design of the decision trees by providing frequency distributions of MPA sizes and inter-MPA distances from which values were chosen in the simulations. We used the policy information from the Fisheries Code to nominate a percentage ceiling of protection of waters at 15% per spatial context. Decision trees were also informed by our key informants, who were asked about the histories of MPAs in the region and the process of establishing them. We ran the decision-tree simulations for 8 years (2012–2020) unless the percentage ceiling was reached in all governance units before 2020. We ran each simulation 100 times, because of the stochastic elements in the decision trees.

For the four systematic scenarios (4–7), we used Marxan [55] to select new MPAs to achieve conservation objectives for habitats. We set objectives for habitats within the governance areas relevant to each scenario (Table 1): 20% of each habitat in each municipality (scenario 4); 20% of each habitat in each alliance (scenario 5); 20% of each habitat in each province (scenario 6); and 20% of each habitat across the Verde Island Passage (scenario 7). For some of the planning units that included the depth class >40 m, we increased costs by multiples of 10 (e.g. 10, 100, 1000) in proportion to distance from the shore to counteract the stochastic element in Marxan that randomly selected from the 14,136 equal-cost planning units containing this depth class. This encouraged Marxan to select suitable planning units closer to the shore, and to increase the compactness of potential MPAs. We ran Marxan for each scenario 100 times, and maximized achievement of conservation objectives by adjusting the species penalty factor. However, we constrained annual contributions to 82.8 km^2^ per year to allow for comparison with the non-systematic scenarios. We assumed that the planning units with highest suitability (lowest cost) in each governance unit would be protected at each annual time step, thereby expanding MPAs in each governance unit at about the same rate.

### Comparisons of expansion scenarios

After all the MPAs had been selected in each expansion scenario, we compared scenarios in terms of achievement of conservation objectives. This involved three kinds of comparisons: 1) between non-systematic scenarios to reflect the influence of different spatial contexts, using objectives set across the whole Verde Island Passage; 2) between systematic scenarios for different spatial contexts, using objectives set across the whole Verde Island Passage; and 3) between non-systematic and systematic scenarios for the same spatial contexts (scenario 1 vs. 4, 2 vs. 5, 3 vs. 6), using objectives set across municipalities, alliances, and provinces, respectively.

For each of these comparisons, we used three sets of calculations. First, across the 100 repeat runs for each simulation, we calculated the average percentage of each habitat protected at each annual time step to determine the efficiency of achieving habitat conservation objectives. More efficient scenarios achieved objectives for habitats sooner, or to a greater degree at the same time step. Second, for each scenario, we calculated the total area protected that contributed to conservation objectives across all habitats at each single time step. This was the sum of the areas protected for all habitats annually, excluding areas in excess of the conservation objectives. Third, we calculated at each time step the total area across all habitats that exceeded the conservation objectives to estimate inefficiency. For each scenario and time step, the sum of the second and third calculations was the total extent of MPAs added. The second and third calculations produced single metrics, aggregated across all habitats, for each scenario to facilitate comparisons.

We also used maps of selection frequencies to compare scenarios spatially. To compare non-systematic and systematic scenarios within the same spatial contexts, we created difference maps by subtracting the selection frequencies of planning units in each non-systematic scenario from those in its corresponding systematic scenario. The difference maps showed which planning units were selected more or less frequently in non-systematic or systematic scenarios. We considered the planning units with selection frequencies >90% as important in any scenario.

## Results

### Suitability layers for the uncoordinated and coordinated scenarios

**Fig 4:**
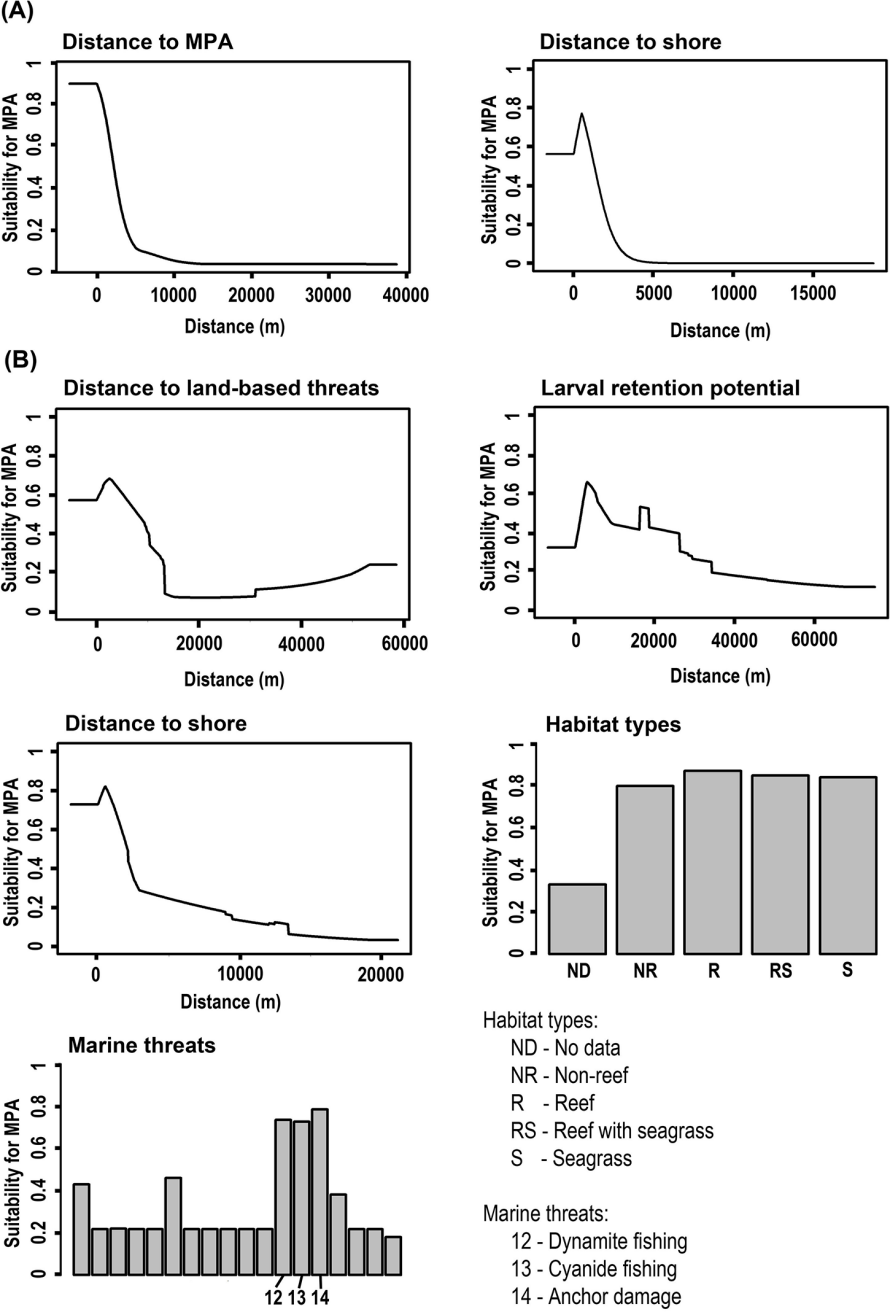
Important predictors of suitability for new MPAs based on Maxent. A) Uncoordinated scenario; B) Coordinated scenarios. The response curves and the bar graphs show the suitability of planning units for MPA establishment in relation to each of the predictors used by the models. These graphs do not incorporate the interactions between the predictors. Distances and categories with suitability values >0.5 indicate potential for MPA establishment in both scenarios.

The suitability layers created for the uncoordinated and coordinated scenarios using Maxent produced good fits to the existing distributions of MPAs in the region (cross-validated AUC values >0.9). Because the predictors used in each of the models were significantly correlated, we investigated the suitability produced by Maxent for each individual predictor in isolation to get an accurate understanding of its influence on the model (Fig 4). Based on the Maxent model for uncoordinated MPAs, the distances to both an existing MPA and the shoreline were good predictors of suitability for new MPAs, contributing 60% and 38% to the explanatory power of the model, respectively. For coordinated MPAs, the most important predictors contributing to the model were: distance to shore (34.7%), distance to land-based threats (27.6%), coastal and marine threats (13.4%), habitat type (13.1%), and potential for larval retention (10%). Suitability was higher in planning units closer to the shoreline for both models (Fig 4).

### Comparison of non-systematic scenarios

**Fig 5:**
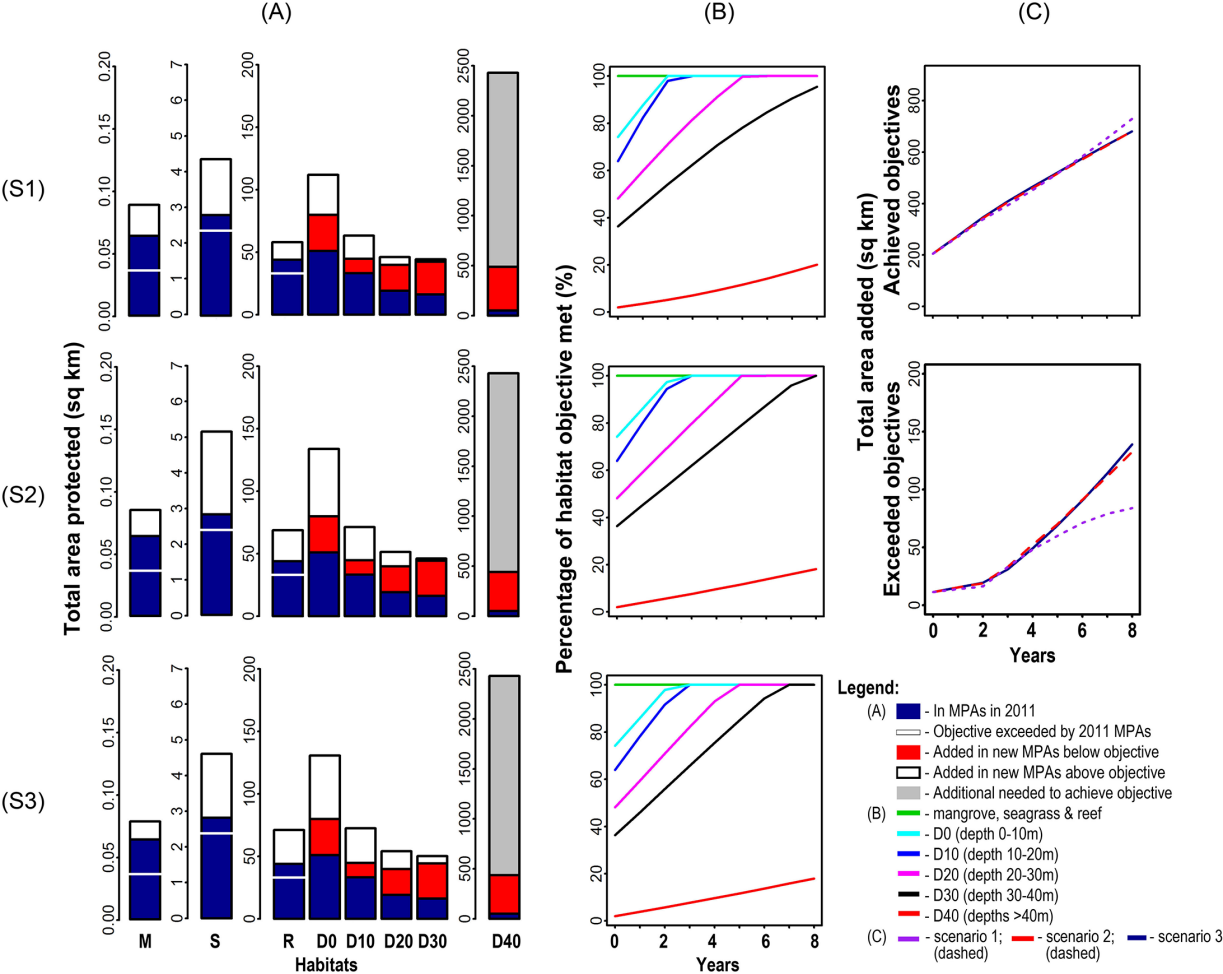
Achievement of objectives for the whole Verde Island Passage in the non-systematic scenarios (1–3). The barplots (A) show the total area of each habitat protected in each scenario (S1-S3) at the end of each simulation (2020). The three line graphs (B) indicate the percentage of objective met for each habitat in each scenario (S1-S3) in each year of the simulation, not counting areas added in excess of objectives. The fourth and fifth line graphs (C) show for each scenario (S1-S3) the total area, summed across habitats, contributing to objectives (top) and exceeding objectives (bottom) in each year of the simulation.

**Fig 6:**
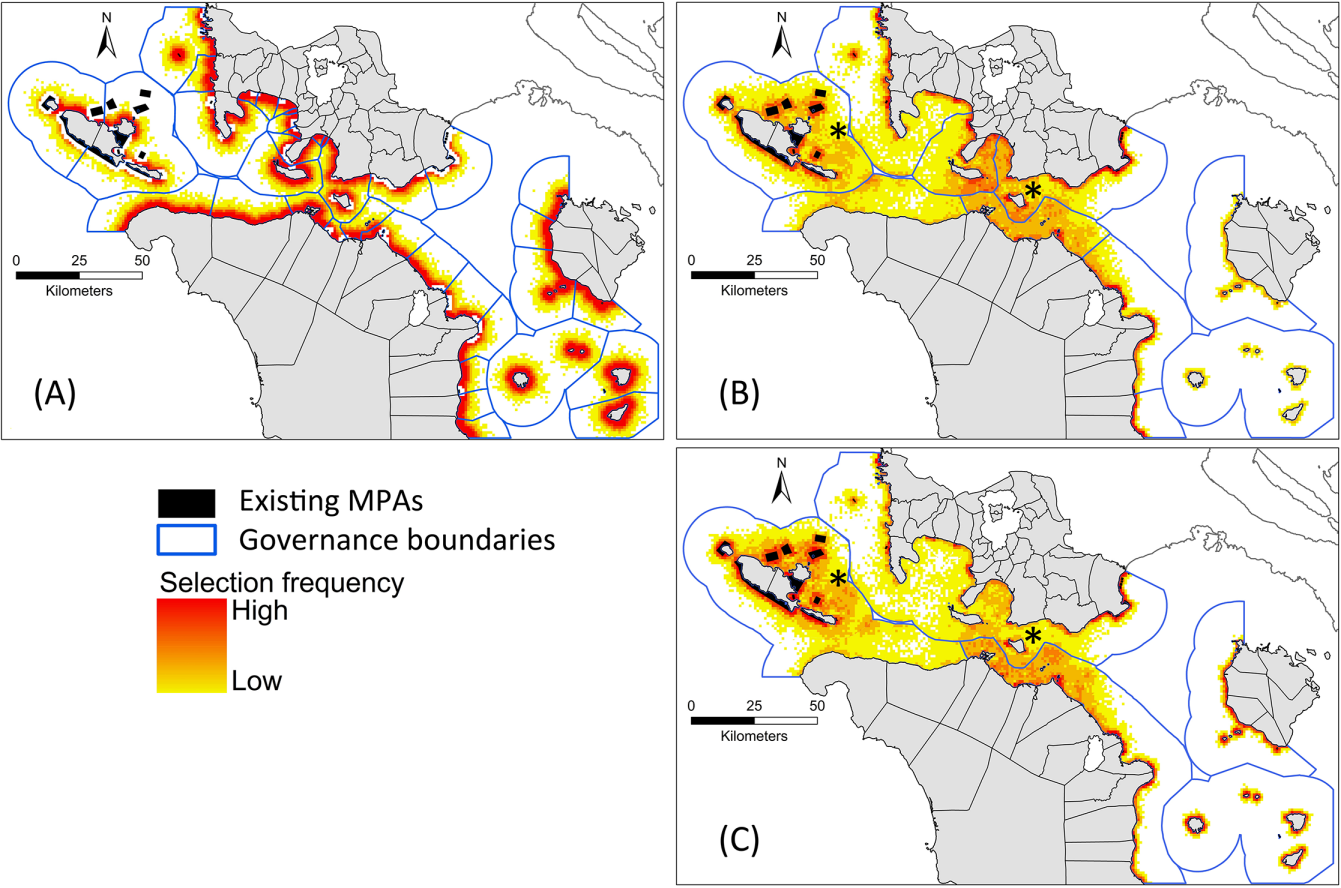
Selection frequencies of planning units across 100 simulation runs for the non-systematic scenarios. A—Scenario 1; B—Scenario 2; C—Scenario 3. Planning units selected more frequently are indicated by warmer colours. Two areas with asterisks are examples of deeper parts of the Passage with higher suitability values in Scenarios 2 and 3.

We found that the existing MPA network (represented by blue bars in Fig 5, and the black polygons in Fig 6) had exceeded the objectives for seagrass, coral reef, and mangrove habitats. The non-systematic scenarios achieved the objectives for most of the remaining habitats by the end of the simulations, but missed the objective for depth class >40m by 80–82% (Fig 5A and 5B). The uncoordinated scenario (1) also missed the objective by 5% for depth class 30–40m. Initial comparison of the three non-systematic scenarios (Fig 5B) showed that the fully coordinated scenario (3) was the most efficient, achieving objectives for most the depth classes 0–30m in years 6–7. Next in efficiency was the partially coordinated scenario (2). However, achievement of objectives for depth >40m was 2% higher in the uncoordinated scenario (1) than in the coordinated scenarios (2–3). The 2% difference led to the uncoordinated scenario (1) contributing the largest total area to objectives (Fig 5C, top), amounting to 44–48 km^2^ more than the coordinated scenarios (2 and 3). Correspondingly, over-achievement of objectives was less for the uncoordinated scenario (1) than for the coordinated scenarios (2 and 3) (Fig 5C, bottom).

Planning units selected more frequently in the uncoordinated scenario (1) were evenly distributed with respect to municipalities and close to the shoreline (Fig 6A). The spread of selections across municipalities can be understood in relation to the suitability layers, decision trees, governance context, and the geomorphology of the Verde Island Passage. The main variables in the suitability layer for uncoordinated scenario were distance to shore and distance to existing MPAs. Hence, the selected planning units were closer to shore and to existing MPAs, causing poorer achievement of objectives for one open-water habitat. In addition, the limit in the decision trees on MPA establishment of 15% of municipal waters, combined with some municipalities having small marine extents and more established MPAs at the beginning of the simulations, led to a more even distribution of MPAs across the municipalities. At the end of the simulations, 31 out of the 36 municipalities had reached the limit of MPA establishment.

Compared to the uncoordinated scenario, planning units selected more frequently in the coordinated scenarios (2 and 3) were less evenly distributed across municipalities but more evenly distributed with respect to distance from shoreline (Fig 6B and 6C). The latter tendency reflected the different factors contributing to suitability for the coordinated scenarios, leading to frequent selection of some planning units close to the shoreline and others in the deeper portions of the Passage (e.g. middle portion and next to Looc municipality marked with an asterisk in Fig 6B and 6C). The limit of 15% of shared municipal waters was not met for any governance areas in the coordinated scenarios because of two factors: the smaller percentages of larger governance areas occupied by existing MPAs; and the larger tracts of combined municipal waters available for protection, making it less likely that annual additions to MPA systems would reach the 15% ceiling.

### Comparison of systematic scenarios

**Fig 7:**
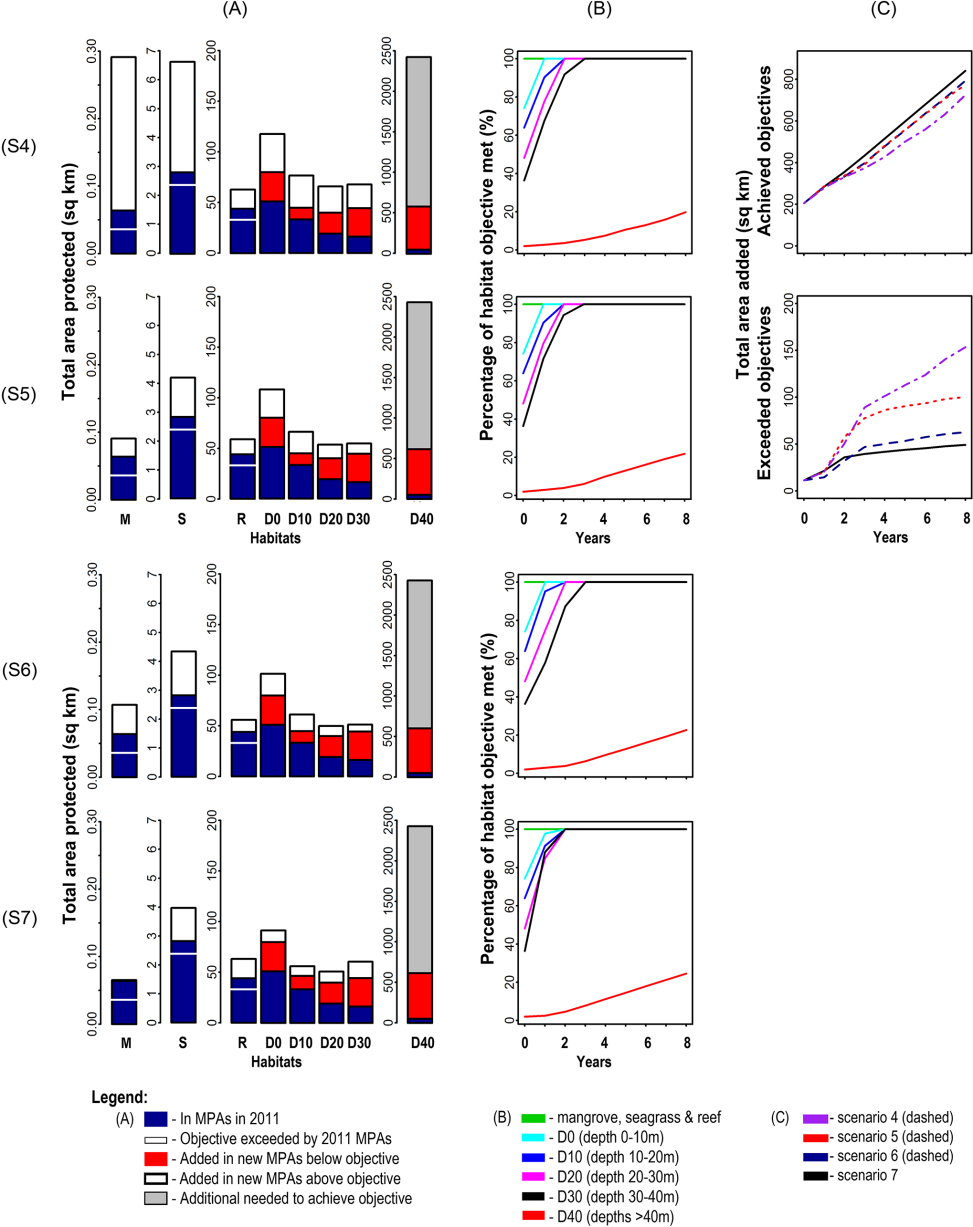
Achievement of objectives for the whole Verde Island Passage in the systematic scenarios (4–7). The barplots (A) show the total area of each habitat protected in each scenario (S4-S7) at the end of each simulation (2020). The four line graphs (B) indicate the percentage of objective met for each habitat in each scenario (S4-S7) in each year of the simulation, not counting areas added in excess of objectives. The fourth and fifth line graphs (C) show for each scenario (S4-S7) the total area, summed across habitats, contributing to objectives (top) and exceeding objectives (bottom) in each year of the simulation.

**Fig 8:**
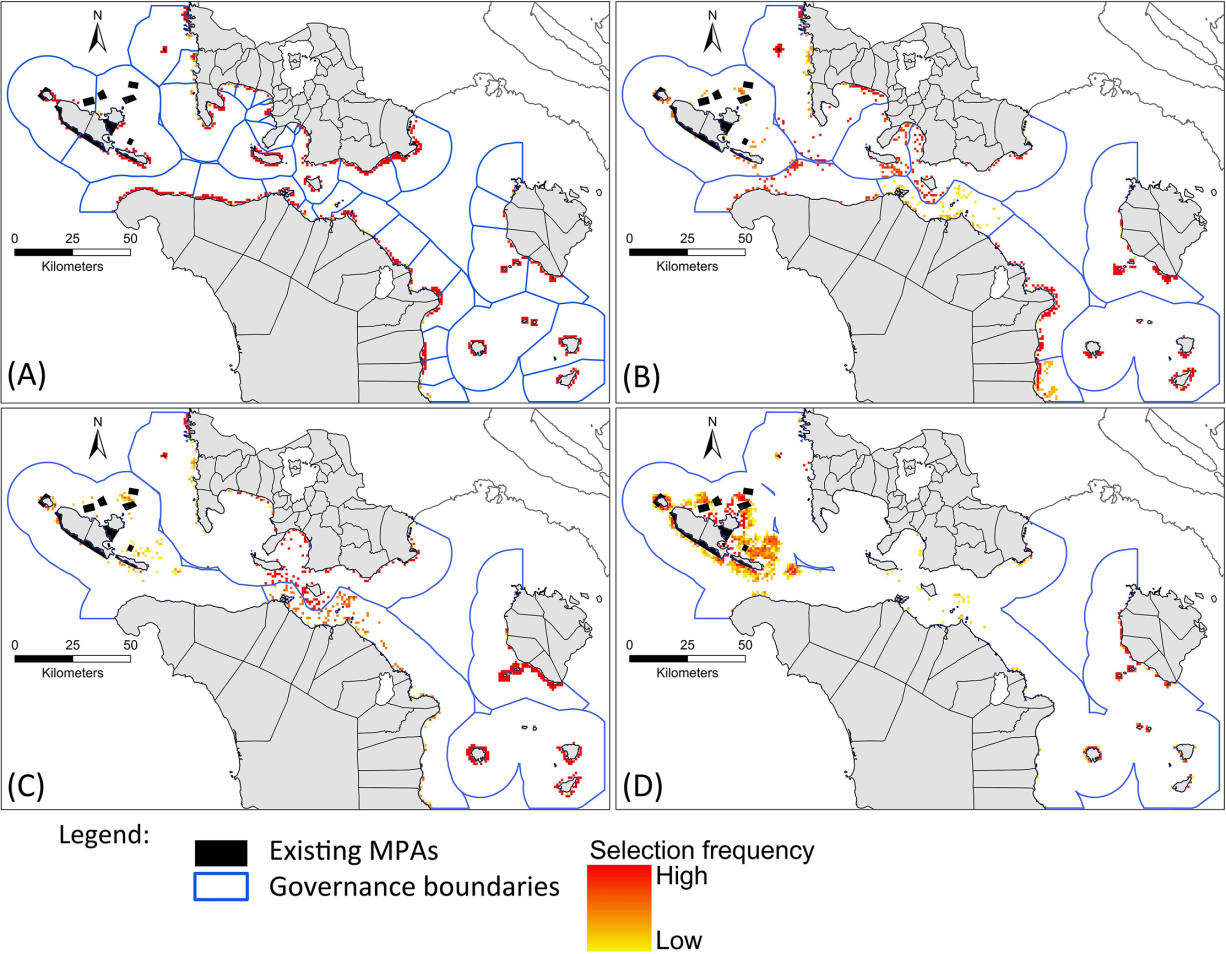
Selection frequencies of planning units across 100 simulation runs for the systematic scenarios. A- Scenario 4; B–Scenario 5; C–Scenario 6; D- Scenario 7. Planning units selected more frequently are indicated by warmer colours.

All the systematic scenarios achieved objectives for all habitats except depth class >40m (Fig 7A). The efficiency of the systematic scenarios varied. The systematic whole-of-region scenario (7) was the most efficient, achieving the objectives for the depth classes 0–30m in year two (Fig 7B) and achieving 24% of the objective for depth class >40m. The remaining systematic scenarios (4–6) achieved objectives for the depth classes 0–30m between years 1 and 4, and achieved 20–22% of the objective for depth class >40m at year 8. The whole-of-region systematic scenario (7) also contributed more total area to achievement of objectives across the Verde Island Passage, with scenarios within progressively narrower governance contexts (6, 5, then 4) contributing progressively less total area (Fig 7C, top). Conversely, scenarios with progressively narrower governance contexts (4, 5, 6 then 7) contributed progressively larger areas in excess of objectives (Fig 7C, bottom). Selection frequencies varied between the systematic scenarios (Fig 8). As the governance boundaries widened from individual municipalities to the whole Verde Island Passage, selection frequencies became less even across municipalities, reflecting the progressive relaxation of spatial constraints on achieving objectives.

### Comparison of non-systematic and systematic scenarios

**Fig 9:**
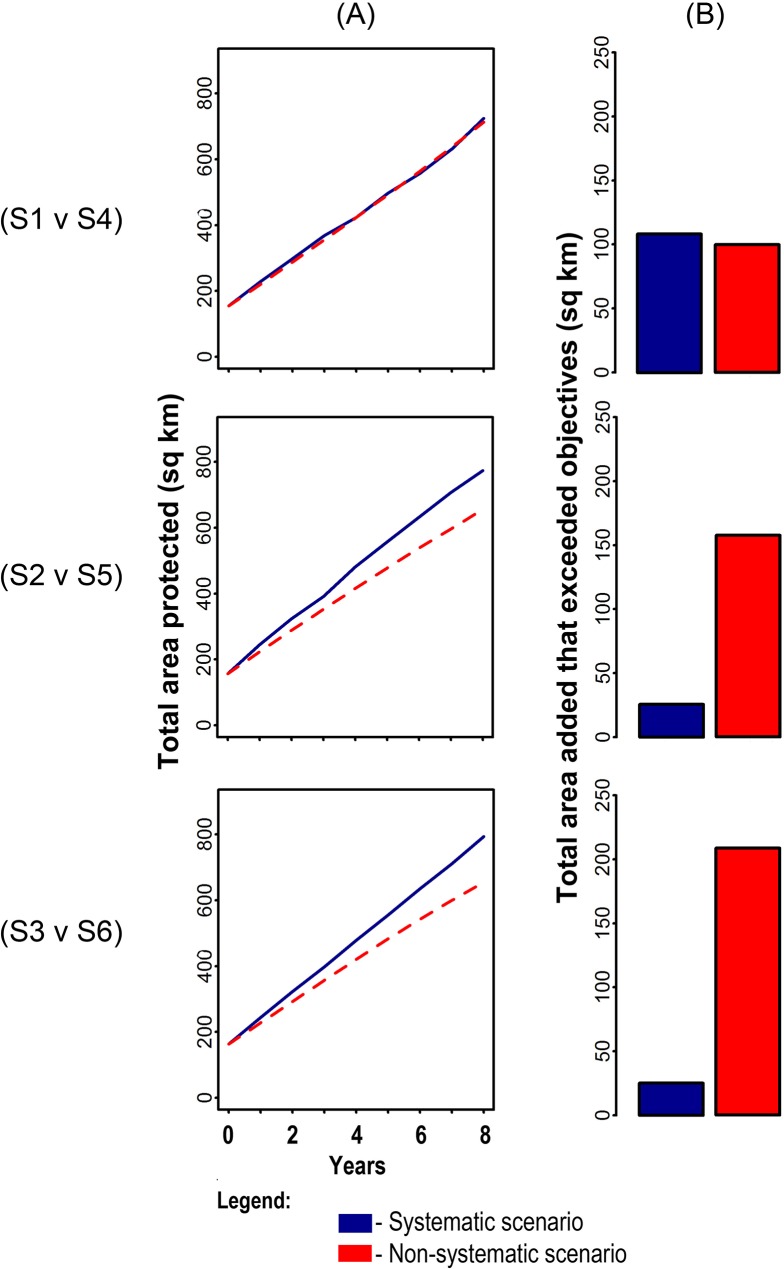
Addition of MPAs relative to habitat conservation objectives by non-systematic and systematic scenarios in three spatial contexts. For both (A) and (B), top graphs are for municipalities, middle graphs for alliances, and bottom graphs for provinces. (A) Comparison of total areas protected across habitats, averaged across the 100 repeat runs, at each annual time step. (B) Comparison of total areas added in excess of objectives across habitats, averaged across the 100 repeat runs, to 2020.

Comparison of the non-systematic and systematic scenarios for each spatial context showed that the systematic scenarios were consistently more efficient in terms of achievement of objectives (Fig 9A). For alliances of local governments, scenario 5 (systematic) contributed ~118 km^2^ more to objectives by 2020 than scenario 2 (non-systematic). For provinces, scenario 6 (systematic) contributed ~184 km^2^ more to objectives than scenario 3 (non-systematic). For municipalities, the difference between scenarios 1 and 4 was slight, with scenario 4 (systematic) contributing about ~11 km^2^ more to objectives than scenario 1 (non-systematic). This over-achievement of objectives in the non-systematic scenarios 2 and 3 was due largely to continuous expansion of MPAs in certain governance areas with higher suitability for establishing MPAs. In contrast to the other systematic scenarios, scenario 4 (municipalities) exceeded objectives by slightly more (~9 km^2^) than scenario 1, its non-systematic equivalent (Fig 9B).

**Fig 10:**
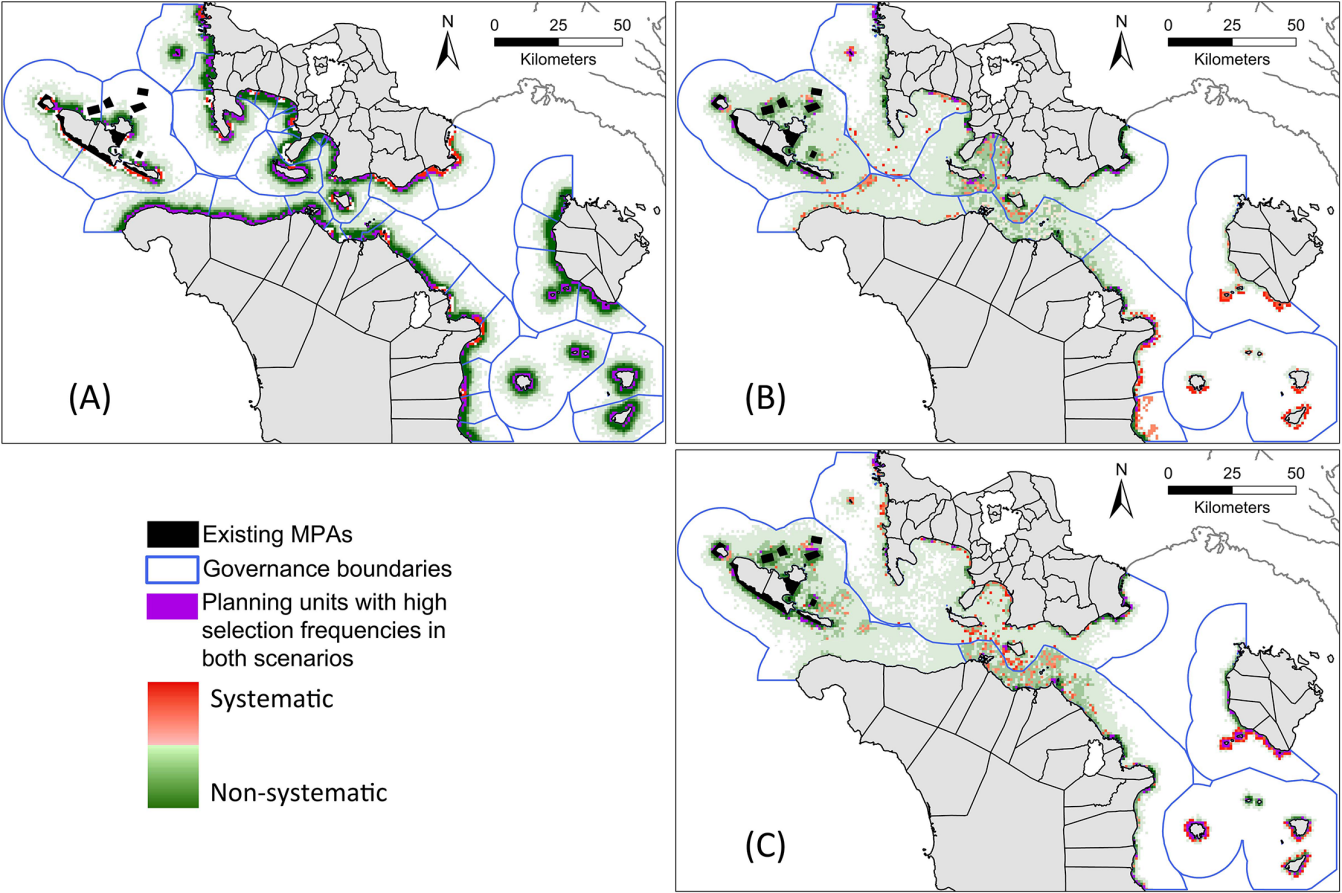
Spatial differences between non-systematic and systematic scenarios applied in the same governance contexts. (A) Selection within 36 municipalities, non-systematic (S1) vs. systematic (S4). (B) Selection within 10 alliances, non-systematic (S2) vs. systematic (S5). (C) Selection within 5 provinces, non-systematic (S3) vs. systematic (S6). Darker red indicates planning units selected more frequently in the systematic scenarios. Darker green indicates planning units selected more frequently in the non-systematic scenarios. Paler colours indicate planning units selected in roughly equal frequency in both non-systematic and systematic scenarios. Purple indicates planning units with selection frequencies >90% in both non-systematic and systematic scenarios.

Spatial comparison of scenarios 1 and 4 (Fig 10A) showed that most planning units along the shoreline were selected frequently in both scenarios. Comparisons of scenarios 2 and 5 (Fig 10B), and scenarios 3 and 6 (Fig 10C) showed that planning units selected more often in the systematic scenarios were located in governance units that had the fewest protected areas, reflecting the influence of conservation objectives. In contrast, the non-systematic scenarios for alliances and provinces selected planning units unevenly between governance units. For alliances and provinces, planning units adjacent to existing MPAs and along the shoreline were selected frequently in both systematic and non-systematic scenarios.

## Discussion

In this paper, we described and compared different approaches to MPA expansion to demonstrate their relative benefits. First, we compared, for both systematic and non-systematic approaches, the effects of governance context on efficiency of achieving regional conservation objectives. Second, we compared the relative benefits of systematic and non-systematic approaches for achieving conservation objectives framed within municipalities, alliances, and provinces.

### Non-systematic scenarios in three governance contexts

Our results contradict our initial assumption that coordination improves establishment of MPAs and has more benefits compared to uncoordinated establishment. However, there is a caveat on the apparent ability of the uncoordinated scenario to better achieve conservation objectives. The simulation for scenario 1 did not reflect all the significant real-world constraints on uncoordinated establishment of MPAs. For comparison with other scenarios, we used a very large annual rate of MPA establishment (82.8 km^2^). Previous studies have shown that the actual annual rate of establishment of uncoordinated community-based MPAs in the Philippines (~1 km^2^) has been insufficient to achieve regional conservation objectives [33, 56]. Moreover, efforts of communities and local governments to establish MPAs have been constrained by institutional capacity, the costs of protecting and managing large areas, and high dependence on fisheries, which has limited the acceptability of MPAs [29, 33, 56]. Hence, realistically, the annual rate of establishment for the uncoordinated, non-systematic scenario should have been smaller, and not all the municipalities would have had MPAs.

Although the non-systematic coordinated scenarios were slightly less efficient than the uncoordinated scenarios, their results reflect the advocacy of Philippine MPA experts, and the potential benefits of to improving design and increasing the rate of MPA expansion. These benefits are possible through the ability of this approach to transcend governance boundaries. An example is the cluster of MPAs in the municipalities of Lubang Island [57]. The annual rate of establishment used for all the scenarios was based on that observed after local governments in the Verde Island Passage began coordinating their efforts. Hence, the coordinated scenarios are more likely to be implemented than the uncoordinated scenario as depicted here. However, we also recognize that the likelihood of implementation of the coordinated scenarios will be limited by the necessary transaction costs (e.g. time and money) [58] of building consensus on the implementation and distribution of MPAs. Moreover, coordinated, regionally-relevant MPAs will not be evenly spread across governance units. Coordination would require local governments and communities to understand and accept that coordinated MPAs come with immediate benefits (e.g. larval spillover) [57] and costs (e.g. forgone fishing) that will not be equitably distributed between municipalities, requiring mechanisms to redistribute costs and benefits in ways agreeable to the parties involved.

### Systematic scenarios in the four governance contexts

The relative efficiencies of the four systematic scenarios accord with results from previous studies [59–64] that demonstrated reductions in efficiencies when selections were constrained within smaller governance contexts. Over-achievement of objectives was higher with smaller governance contexts because representation was repeated to achieve objectives in each geographic area. Although, in principle, 20% of a habitat across the Verde Island Passage is the same amount as 20% of each habitat in each municipality, the inevitable inefficiencies of representation within 1 km^2^ planning units were repeated more often within narrower spatial contexts, leading to less efficient use of MPAs. In our study, however, the differences in efficiency between systematic selections in the different contexts were reduced by all scenarios having the same annual rate of establishment of MPAs and the same total allocated area over eight years. In contrast, previous studies have identified the total cost (in extent or funds) needed to achieve all objectives in different contexts, allowing influence of governance contexts to be fully expressed.

### Systematic vs. non-systematic approaches

In the context of municipalities, alliances, and provinces, the systematic scenarios were more efficient at achieving objectives than their non-systematic counterparts. These broad results were expected, and in line with previous studies [6, 38, 65, 66], because the systematic selections were directed primarily at achieving objectives with complementarity between newly-selected MPAs while also recognising the contributions to objectives of MPAs established before the simulations began. In contrast, the non-systematic scenarios were guided by rules in the decision trees that did not address objectives and by suitability layers that either ignored habitats (for municipalities) or were only slightly influenced by habitat types (alliances and provinces). The enhanced efficiency of the systematic scenarios was substantial for alliances and provinces, for which over-achievement of objectives was higher in the non-systematic scenarios, as expected from previous studies.

Contrary to expectations, within municipal boundaries, over-achievement of objectives was higher for the systematic than the non-systematic scenario. Further, the increased efficiency of the systematic scenario was negligible. This finding contrasts with that of Mills, Adams (38), who observed large differences between non-systematic and systematic selections in the context of local governance units, effectively our scenarios 1 and 4, respectively. There were three reasons for these contrasting results. First, because our non-systematic scenarios often selected parts of planning units, the effective average size of planning units was smaller (S1 and S2 Tables). Consequently, incidental representation [67] was reduced and efficiency increased relative to the corresponding systematic scenario that used whole planning units (and this factor would have narrowed the gap in efficiency between all pairs of systematic and non-systematic scenarios). The second reason was the 15% limit, in the non-systematic scenario, on MPAs in any one local government area, causing MPAs to be spread across municipalities and to contribute to objectives more effectively than the other non-systematic scenarios (2 and 3). The third reason was the different use of suitability layers in this study and that of Mills, Adams (38). Although our non-systematic scenarios were not directed at achieving objectives, the suitability layer and decision tree for the simulation within municipalities selected planning units close to the shoreline and to existing MPAs, leading to protection of fringing habitats and shallower depths, similar to the corresponding systematic scenario. In contrast, the non-systematic scenario of Mills, Adams (38) protected areas without mapped habitats because their predictors of suitability included, as well as habitats and distance measures, proportion of fishing ground closed and presence of provincial management support teams.

### Other caveats and future directions

Given that we did not have data on the relative effectiveness of the different zones for protecting different species and habitat types, we were not able to describe the trade-offs associated with different forms of MPA management in the scenarios. Based on the MPA data and the interviews with the local managers, there might have been preferences for different MPA zones in the uncoordinated and coordinated scenarios. We suggest that this be included in further studies to determine if the preferences for different MPA zones would lead to more divergent achievement of conservation objectives. Moreover, the results of these kinds of scenarios could also be compared in the future with a broader range of objectives, including those related to fisheries and connectivity.

## Conclusion

This study builds on the work of Mills, Adams (38), which compared systematic selections against a realistic, non-systematic approach. Mills, Adams (38) suggested that partial coordination of local decisions about MPAs would be intermediate in efficiency between uncoordinated local decisions and region-wide systematic planning. Our findings do not support this suggestion, because the non-systematic, uncoordinated scenario was more efficient than either of the coordinated scenarios. However, our non-systematic uncoordinated scenario could also be regarded as unrealistic, and its results overly optimistic, because the rate of expansion used in the simulations were based on the MPAs established during the period of coordination. Hence, we believe that, despite our results, the coordinated scenarios have the potential to be more efficient than the uncoordinated scenario. Moreover, although we did not explore coordinated local decisions that were directed, fully or partially, at achieving conservation objectives, the higher efficiency of systematic scenarios in the context of provinces, alliances, and municipalities suggests that coordination of local actions focused on explicit objectives would at least partly support the prediction of Mills, Adams (38) in our study region. We suggest that coordinated planning using systematic approaches be used in the study region, and in other areas with similar, local devolution of decision-making, to better achieve conservation goals framed across broad contexts.

## Supporting Information

S1 Fig Scenario 1—Uncoordinated MPA establishment decision tree. This decision tree describes the steps taken to simulate uncoordinated community and/or locally-based MPA establishment. A single municipality can establish one or more MPAs in a single year, provided that it does not exceed the percentage area allowed for MPAs based on the Fisheries Code. White boxes present alternate routes. Explanations for main steps are detailed in S1 Table.
(DOCX)

S2 Fig Scenarios 2 and 3—Partially and Fully Coordinated Scenarios decision tree. This decision tree presents the steps taken during two levels of coordinated MPA establishment. An alliance of two or more municipalities (Scenario 2) and all municipalities within provinces (Scenario 3) can establish one or more MPAs in a single year, provided that they do not exceed the percentage area allowed for MPAs based on the Fisheries Code. White boxes present alternate routes. Both alliances and provinces are referred to here as “clusters” of municipalities. Explanations for main steps are found in S2 Table.
(DOCX)

S1 Table Scenario 1. Uncoordinated MPA establishment and rationale for main decision steps. The rationale was based on interview data, policy information, and MPA databases.
(DOCX)

S2 Table Scenarios 2 and 3. Two levels of coordinated MPA establishment and rationale for main decision steps. The rationale was based on interview data, policy information, and MPA databases.
(DOCX)
