# Supplementary material for: Benefits and Challenges of Scaling Up Expansion of Marine Protected Area Networks in the Verde Island Passage, Central Philippines
Source: PLoS One. 2015 Aug 19;10(8):e0135789. doi: 10.1371/journal.pone.0135789 (PMC4545830; doi:10.1371/journal.pone.0135789)
Supplement: S1 Fig — This decision tree describes the steps taken to simulate uncoordinated community and/or locally-based MPA establishment. A single municipality can establish one or more MPAs in a single year, provided that it does not exceed the percentage area allowed for MPAs based on the Fisheries Code. White boxes present alternate routes. Explanations for main steps are detailed in S1 Table. (DOCX) [file pone.0135789.s001.docx]

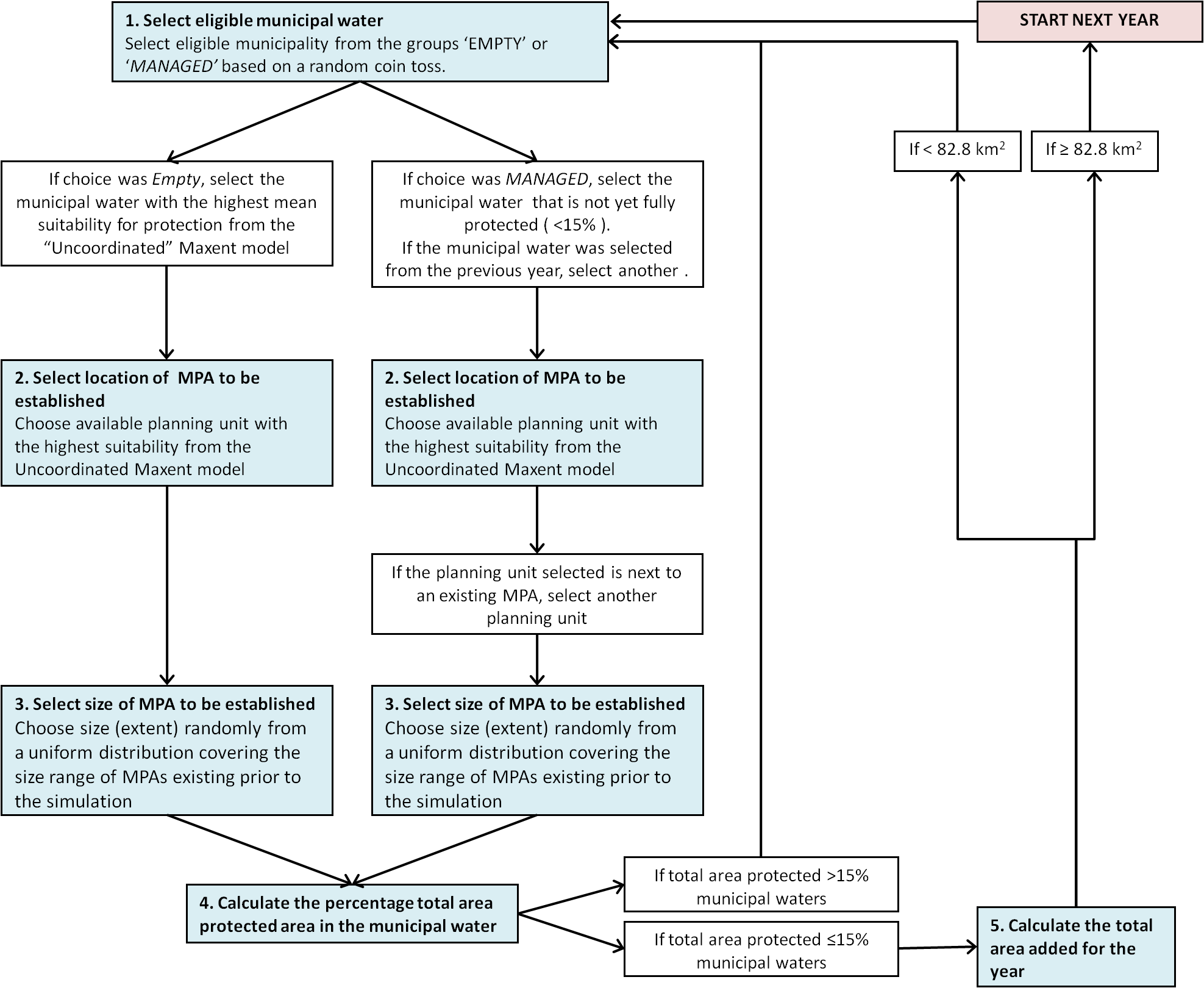


**Figure S1. Scenario 1 - Uncoordinated MPA establishment decision tree. This decision tree describes the steps taken to simulate uncoordinated community-based MPA establishment.** A single municipality can establish one or more MPAs in a single year, provided that it does not exceed the percentage area allowed for MPAs based on the Fisheries Code. White boxes present alternate routes. Explanations for main steps are detailed in Table S3. MatLab codes are available upon request.
