# Supplementary material for: Benefits and Challenges of Scaling Up Expansion of Marine Protected Area Networks in the Verde Island Passage, Central Philippines
Source: PLoS One. 2015 Aug 19;10(8):e0135789. doi: 10.1371/journal.pone.0135789 (PMC4545830; doi:10.1371/journal.pone.0135789)
Supplement: S2 Fig — This decision tree presents the steps taken during two levels of coordinated MPA establishment. An alliance of two or more municipalities (Scenario 2) and all municipalities within provinces (Scenario 3) can establish one or more MPAs in a single year, provided that they do not exceed the percentage area allowed for MPAs based on the Fisheries Code. White boxes present alternate routes. Both alliances and provinces are referred to here as “clusters” of municipalities. Explanations for main steps are found in S2 Table. (DOCX) [file pone.0135789.s002.docx]

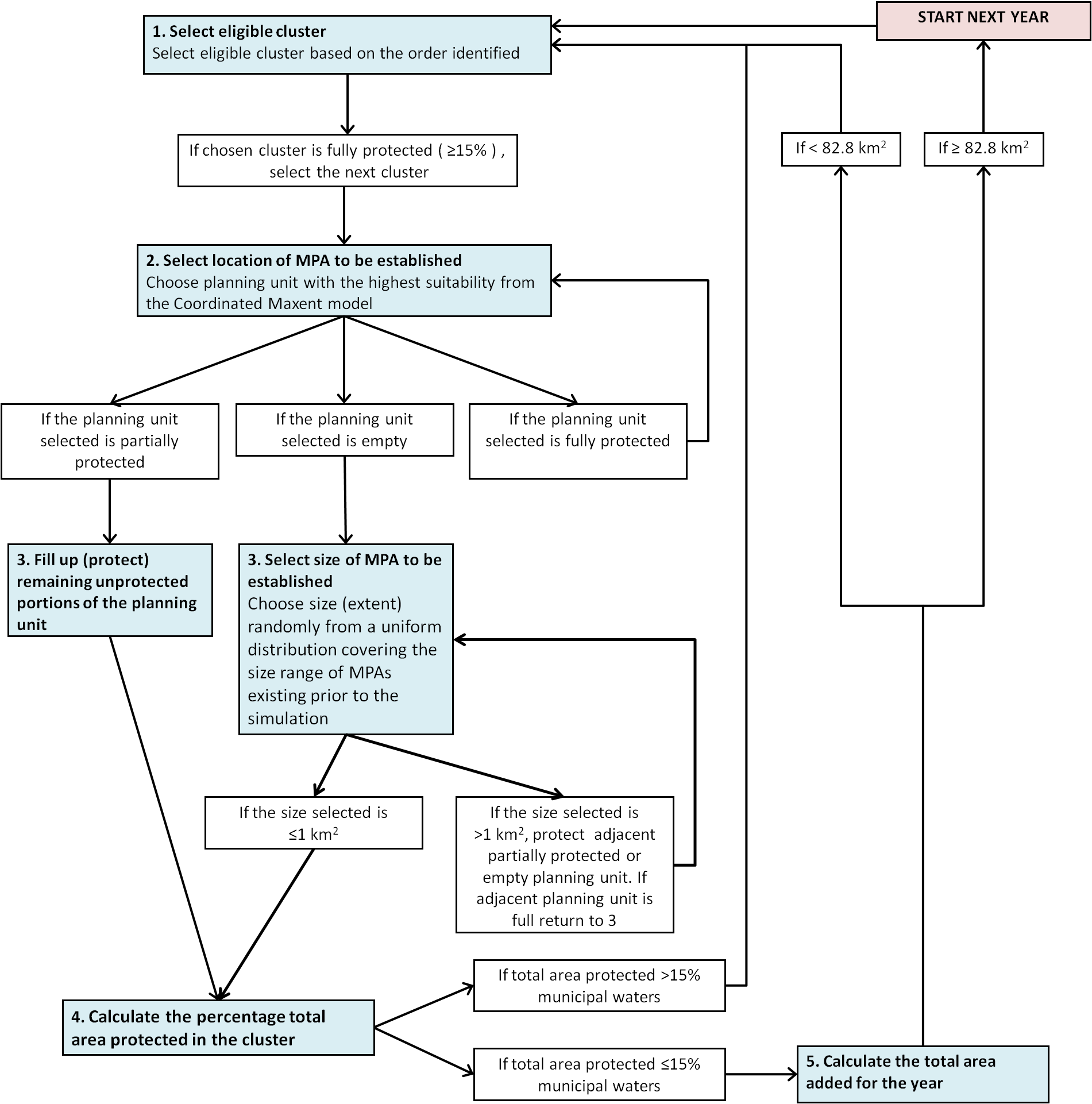


**S2 Fig. Scenarios 2 and 3 - Partially and Fully Coordinated Scenarios decision tree. This decision tree presents the steps taken during two levels of coordinated MPA establishment.** An alliance of two or more municipalities (Scenario 2) and all municipalities within provinces (Scenario 3) can establish one or more MPAs in a single year, provided that they do not exceed the percentage area allowed for MPAs based on the Fisheries Code. White boxes present alternate routes. Both alliances and provinces are referred to here as “clusters” of municipalities. Explanations for main steps are found in Table S4. MatLab codes are available upon request.
