# Supplementary material for: Benefits and Challenges of Scaling Up Expansion of Marine Protected Area Networks in the Verde Island Passage, Central Philippines
Source: PLoS One. 2015 Aug 19;10(8):e0135789. doi: 10.1371/journal.pone.0135789 (PMC4545830; doi:10.1371/journal.pone.0135789)
Supplement: S1 Table — The rationale was based on interview data, policy information, and MPA databases. (DOCX) [file pone.0135789.s003.docx]

**S1 Table. Scenario 1. Uncoordinated MPA establishment and rationale for main decision steps.**

The rationale was based on interview data, policy information, and MPA databases.

| **Steps** | **Rationale** |
| --- | --- |
| *Step 1. Select eligible municipal water* | We used a coin toss to select a group of local governments, because we assumed there are equal chances of an MPA being established in municipal waters that have MPAs (MANAGED) or not (EMPTY). Although each local government was urged to have an MPA in its area, depending on the beliefs and objectives of the chief executives and communities, some municipalities have none and others have more than one. We capped protection to 15% of the total area of municipal waters based on the mandate of The Philippine Fisheries Code. We used the uncoordinated suitability model to inform our choice of municipality without existing MPAs because we assumed that municipalities that are most suitable overall will be the most willing to accept new MPAs. |
| *Step 2. Select location of MPA to be established* | The local government and community select areas for MPA establishment, after a baseline assessment and/or series of public consultations. We used the Maxent model as a surrogate for the baseline assessment, assuming that the planning units with the highest modelled suitability were most likely to have MPAs established. The new MPAs will be at least 1 km away from the nearest MPA to allow space for subsistence fishers. We selected that minimum distance since we assumed, to best represent the actual situation, that MPAs would still be close to each other and to the shoreline for ease of enforcement. |
| *Step 3. Select size of MPA to be established* | The local government and community compromise on the total area to be protected. This is done through a series of public consultations. We used the size range of community-based MPAs (min = 0.02 km^2^; max = 0.90 km^2^; median = 0.12 km^2^), established prior to coordination, to inform our selection so we could best represent reality. |
| *Step 4. Calculate the total percentage of water protected* | Local governments are responsible for zoning their municipal waters (e.g. areas to be utilized for fishing, shipping lanes). We assumed more than one MPA would be established within a single municipal water area within one year (below). However, we limited protection to just 15% of the total municipal water. If the size selected contributed to excess protection, the model went back to Step 1 to select another municipal water. |
| *Step 5. Calculate the total area added for the year* | Local governments and communities decide to establish MPAs at different barangays (villages) within the municipality nearly at the same time. We assumed more than one MPA would be established within a single municipal water within one year. Hence, if the average annual rate of establishment (e.g. 82.8 sq km) was not achieved, protection in other areas or the same municipal water was allocated. |
