# Supplementary material for: Benefits and Challenges of Scaling Up Expansion of Marine Protected Area Networks in the Verde Island Passage, Central Philippines
Source: PLoS One. 2015 Aug 19;10(8):e0135789. doi: 10.1371/journal.pone.0135789 (PMC4545830; doi:10.1371/journal.pone.0135789)
Supplement: S2 Table — The rationale was based on interview data, policy information, and MPA databases. (DOCX) [file pone.0135789.s004.docx]

**S2 Table. Scenarios 2 and 3. Two levels of coordinated MPA establishment and rationale for main decision steps.**

The rationale was based on interview data, policy information, and MPA databases.

| **Steps** | **Rationale** |
| --- | --- |
| *Step 1. Select eligible shared municipal waters* | Local governments in clusters (within alliances or provinces) collectively decide to establish MPAs in their shared municipal waters. Sharing municipal waters can potentially increase the sizes of their MPAs. Some clusters have had support from bridging organizations and some have not. Partnering with a bridging organization (e.g. NGO) makes clusters work more efficiently. We assumed that the clusters that benefited from external support gained a lot of experience, and were already working well together. These alliances were then were able establish MPAs more efficiently and faster compared to other alliances. We created an order of selection based on the uncoordinated suitability model and interviews. We assumed that alliances that were most suitable overall and had more experience would be the most willing to establish new MPAs. We used the same limit to shared municipal waters as in the uncoordinated scenario, so we capped additions of MPAs at 15% of the total shared municipal waters. |
| *Step 2. Select location of MPA to be established* | The cluster selects the area for MPA establishment, after a baseline assessment. A series of public consultations and field visits with their respective communities are undertaken to decide whether expansion of existing MPAs is possible or new MPAs can be established. Fisher representatives are taken on site visits to discuss the locations and boundaries of the MPAs. We used the Maxent model as a surrogate for the planning process for MPA expansion, assuming that the planning units from the Maxent model with the highest suitability were most suitable for MPA establishment. We assumed that planning units that were only partially protected would have MPAs expanded to occupy their full extents. We assumed that protection of empty planning units adjacent to protected planning units constituted expansion of the adjacent MPA. In contrast, establishment of MPAs in empty planning units next to other empty planning units constituted new, not expanded, MPAs. |
| *Step 3. Select size of MPA to be established* | The clusters and their communities compromise on the total area to be protected. This is done through a series of public consultations. . If the planning unit is partially protected, the planning unit will be filled up to protect the entire planning unit. However, if the planning unit selected was empty, the size of the MPA was selected from the size range of coordinated MPAs (min = 0.01 km^2^; max = 1.71 km^2^; median = 0.41 km^2^), to inform our selection so we could best represent reality Figure S2 shows that, if the selected size of MPA in the simulation was > 1km^2^, part of the adjacent planning unit was also allocated to MPA, if possible. |
| *Step 4. Calculate the total percentage of shared waters protected* | Local governments within a cluster coordinate zoning of their municipal waters (e.g. areas to be utilized for fishing, shipping lanes). We assumed more than one MPA would be established within a cluster within one year. However, we limited protection to just 15% of the total municipal waters in a cluster. If the size selected contributed to excess protection, the model went back to Step 1 and selected the next shared municipal water. |
| *Step 5. Calculate the total area added for the year* | Local governments within a cluster in consultation with their communities decide to establish MPAs at different barangays (villages) within clusters nearly at the same time. We assumed more than one MPA would be established within a cluster within one year. Hence, if the average annual rate of establishment (e.g. 82.8 sq km) was not achieved, protection in a different cluster was allocated. |
